# Supplementary material for: Samples and data accessibility in research biobanks: an explorative survey
Source: PeerJ. 2016 Feb 25;4:e1613. doi: 10.7717/peerj.1613 (PMC4782685; doi:10.7717/peerj.1613)
Supplement: File S1 [file peerj-04-1613-s001.pdf]

Samples and data accessibility of population biobanks: an explorative survey

Marco Capocasa[1*], Paolo Anagnostou[1,2*], Flavio D'Abramo[3*], Giulia Matteucci[1], Valentina Dominici[1,2], Giovanni Destro Bisol[1,2] and Fabrizio Rufo[1,2]

[1]Istituto Italiano di Antropologia, Rome, Italy
[2]Department of Environmental Biology, Sapienza University of Rome, Rome, Italy
[3]Charité Comprehensive Cancer Center, Berlin, Germany

Supplementary File S1: *The questionnaire*

A. General information

A1. Name of Biobank

A2. Biobank Country

A3. The biobank is funded by:
- public funds
- private funds
- both

A4. Which are the sampling criteria used to collect samples?
- geographic
- cultural
- disease
- tissue
- other (specify)

A5. The biobank stores:
- only biological samples
- both biological samples and data

B. Biological samples

B1. Which kind of biological samples (e.g. tissues, cell lines, DNA) are stored in the biobank?

B2. Which are the ethical requirements followed by the biobank in the sample collection procedure?

B3. Does the biobank also store biological samples collected by external research groups?
- yes
- no

B3.1 If yes, do they have to follow the same ethical requirements used by the biobank?
- yes
- no
- other (specify)

B4. Biological samples stored in the biobank are:
- freely accessible
- not accessible
- conditionally accessible

B4.1 If sample are conditionally accessible, which are the accessibility criteria adopted by the biobank?

B5. Does the biobank refer to a specific legal framework for the access to the biological samples?
- yes
- no

B5.1 If yes, which is this legal framework?

B5.2 If yes, where it is possible to find it?

C. Data

C1. The biobank stores:
- only data produced by its own research staff
- also data produced by other research groups that use the biobanks' samples

C2. Data stored in the biobank are:
- freely accessible
- not accessible
- conditionally accessible

C2.1 If data are conditionally accessible, which are the accessibility criteria adopted by the biobank?

C3. Does the biobank refer to a specific legal framework for the data access?

C3.1 If yes, which is this legal framework?

C3.2 If yes, where it is possible to find it?

C3.3 If the biobank also stores data produced by other research groups, are these subject to the same legal framework?
- yes
- no
